# Aquiluscidin, a Cathelicidin from *Crotalus aquilus*, and the Vcn-23 Derivative Peptide, Have Anti-Microbial Activity against Gram-Negative and Gram-Positive Bacteria

**DOI:** 10.3390/microorganisms11112778

**Published:** 2023-11-15

**Authors:** Edwin Esaú Hernández-Arvizu, Teresa Monserrat Silis-Moreno, José Alejandro García-Arredondo, Angelina Rodríguez-Torres, José Antonio Cervantes-Chávez, Juan Mosqueda

**Affiliations:** 1 Immunology and Vaccine Research Laboratory, Natural Sciences College, Autonomous University of Queretaro, Queretaro 76230, Mexico; esau.hernandez@uaq.mx (E.E.H.-A.);; 2 Ph.D. Program in Natural Sciences, Natural Sciences College, Autonomous University of Queretaro, Queretaro 76230, Mexico; 3 Departamento de Investigación Química y Farmacológica de Productos Naturales, Facultad de Química, Universidad Autónoma de Querétaro, Centro Universitario S/N, Queretaro 76010, Mexico; alejandro.gr@uaq.mx; 4 Natural Sciences College, Autonomous University of Querétaro, Queretaro 76230, Mexico; angelina@uaq.mx (A.R.-T.); jose.antonio.cervantes@uaq.mx (J.A.C.-C.); 5 Cuerpo Academico Salud Animal y Microbiología Ambiental, Autonomous University of Queretaro, Queretaro 76230, Mexico

**Keywords:** cathelicidin, anti-microbial peptide, *Crotalus aquilus*, Gram-positive bacteria, Gram-negative bacteria

## Abstract

Anti-microbial peptides play a vital role in the defense mechanisms of various organisms performing functions that range from the elimination of microorganisms, through diverse mechanisms, to the modulation of the immune response, providing protection to the host. Among these peptides, cathelicidins, a well-studied family of anti-microbial peptides, are found in various animal species, including reptiles. Due to the rise in anti-microbial resistance, these compounds have been suggested as potential candidates for developing new drugs. In this study, we identified and characterized a cathelicidin-like peptide called Aquiluscidin (Aq-CATH) from transcripts obtained from the skin and oral mucosa of the Querétaro’s dark rattlesnake, *Crotalus aquilus*. The cDNA was cloned, sequenced, and yielded a 566-base-pair sequence. Using bioinformatics, we predicted that the peptide precursor contains a signal peptide, a 101-amino-acid conserved cathelin domain, an anionic region, and a 34-amino-acid mature peptide in the C-terminal region. Aq-CATH and a derived 23-amino-acid peptide (Vcn-23) were synthesized, and their anti-microbial activity was evaluated against various species of bacteria in in vitro assays. The minimal inhibitory concentrations against bacteria ranged from 2 to 8 μg/mL for both peptides. Furthermore, at concentrations of up to 50 μM, they exhibited no significant hemolytic activity (<2.3% and <1.2% for Aquiluscidin and Vcn-23, respectively) against rat erythrocytes and displayed no significant cytotoxic activity at low concentrations (>65% cell viability at 25 µM). Finally, this study represents the first identification of an antimicrobial peptide in *Crotalus aquilus*, which belongs to the cathelicidin family and exhibits the characteristic features of these peptides. Both Aq-CATH and its derived molecule, Vcn-23, displayed remarkable inhibitory activity against all tested bacteria, highlighting their potential as promising candidates for further antimicrobial research.

## 1. Introduction

Anti-microbial resistance is a global medical emergency that poses a significant risk to human and animal health, leading to increased diseases caused by resistant microorganisms [1]. It has become a top priority in the medical field. The rampant and indiscriminate use of antibiotics and other anti-microbial compounds has accelerated the emergence of resistance [1]. In response to this urgent situation, extensive research is being conducted to discover new molecules to address this problem [2]. Alarming reports suggest that by 2050, approximately 10 million people could die from infections caused by antibiotic-resistant microorganisms [3].

Anti-microbial peptides (AMPs), also called host defense peptides (HDPs), are innate immune response molecules known for their broad-spectrum activity against various classes of microorganisms [4,5]. They can exert their activity by directly killing microorganisms or by carrying out actions that stimulate the host’s immune responses [5,6]. Among the microbicidal mechanisms of action of AMPs are membrane disruption, prevention of cell wall formation, and inhibition of protein synthesis and folding [5,6,7]. This unique characteristic has spurred research into their potential as therapeutic alternatives [4,8,9]. AMPs are synthesized by leukocytes and epithelial cells in different organs [10]. Cathelicidins, a prominent family of AMPs, have been identified in various animal species, including mammals, birds, fishes, and reptiles [7,11,12,13]. These peptides are initially produced as pro-peptides and are characterized by the presence of a cathelin domain and a protease cleavage site, which is essential for the release of the mature peptide responsible for the anti-microbial activity [14,15]. To overcome limitations, derivative peptides and analogs have been designed as cost-effective options with reduced undesirable effects such as hemolytic and cytotoxic activity while maintaining their anti-microbial efficacy [16,17,18,19,20].

Snake cathelicidins have been sourced either from the venom gland [21,22,23] or isolated directly from venom [19]. Evaluation of these peptides has demonstrated their high effectiveness against bacteria [24], fungi [25], and parasites [26]. Moreover, derived peptides have also exhibited noteworthy efficacy [18,27].

*Crotalus aquilus*, commonly known as Queretaro’s dark rattlesnake, is an endemic viperid species found in the state of Queretaro, with a wide distribution across central Mexico [28]. Prior to this study, no AMPs had been identified in this particular species. In this study, we present the first identification of the cathelicidin peptide from the *Crotalus aquilus*, named Aquiluscidin. Furthermore, we selected a shorter region, Vcn-23, to evaluate its anti-microbial activity. We assessed the anti-microbial effects of both Aquiluscidin and Vcn-23 against Gram-positive and Gram-negative bacterial strains. Additionally, we examined these peptides’ potential hemolytic and cytotoxic activities on mammalian cells.

## 2. Materials and Methods

All procedures and experimental protocols were evaluated and approved by the Bioethics Committee of the College of Natural Sciences at the Autonomous University of Querétaro, with registration number 38FCN2017. The specimens of the species *Crotalus aquilus* used in this project belong to the collection of the Herpetario Mundo Reptilia, located in Santa Rosa Jauregui, Querétaro, Mexico.

### 2.1. Microorganisms

Six laboratory strains of bacteria were evaluated: *E. coli* (commercial TOP10), *P. aeruginosa*, *S. aureus* ATCC 6538, *S. saprophyticus* ATCC BAA-750, *Salmonella typhimurium* ATCC 14028, and *Enterococcus casseliflavus* ATCC 700327. These strains were provided by the Laboratory of Molecular Biology of Microorganisms at the Autonomous University of Querétaro. Additionally, strains of *P. aeruginosa*, *E. coli*, and *S. aureus* obtained from human clinical isolates were donated by the Regional Hospital # 1 of the Mexican Institute of Social Security.

### 2.2. Sample Collection

Oral mucosa and skin biopsies were obtained from an adult female *Crotalus aquilus* rattlesnake at the Small Species Veterinary Hospital of the School of Natural Sciences at the Autonomous University of Querétaro. The surgical procedure was conducted under anesthesia administered to the specimen. Before induction, atropine sulfate (0.01–0.04 mg/kg IC) was administered for 15 min [29]. Anesthesia induction and maintenance were performed using isoflurane (2–4%) [30], with a maintenance level of 1.5%. In the dorsolateral region, lidocaine (5 mg/kg) [29] was infiltrated, and a 1.5 cm^2^ skin fragment was obtained. The skin was sutured in an eversion pattern using polyglycolic acid [30]. The oral mucosa was gently scraped using a sterile scalpel blade to obtain small tissue samples. Both the skin and oral mucosa samples were placed in TRIzol reagent (Invitrogen^TM^, Carlsbad, CA, USA) at a ratio of 50–100 mg tissue/mL TRIzol and transported frozen to the laboratory, where they were stored at −78 °C. Antibiotic treatment (amikacin 2–4 mg/kg every 72 h intramuscular (IM), three applications) and analgesic treatment (ketoprofen 2–4 mg/kg every 48 h IM) were administered [29].

### 2.3. cDNA Cloning and Sequencing

Total RNA was extracted using the EZ-10 Spin Column Total RNA Mini-Preps Kit (BIO BASIC, Amherst, NY, USA) following the manufacturer’s instructions. Subsequently, it was treated with DNAse I Amplification Grade (Invitrogen^TM^, Carlsbad, CA, USA), according to the protocol provided by the manufacturer. The cDNA was synthesized using the SuperScript III First-Strand Synthesis System for RT-PCR Kit (Invitrogen^TM^, Carlsbad, CA, USA) with OligodT (included in the kit) and gene-specific primers.

A multiple alignment of sequences from the Nucleotide database of the National Center for Biotechnology Information was performed to compare cathelicidins from elapids such as *B. fasciatus*, *O. hannah*, *N. atra* [19,23], and South American vipers including *C. durissus*, *L. muta*, *B. atrox*, and *B. lutzi* [21]. The alignment was carried out using the EMBL-EBI MUSCLE server (https://www.ebi.ac.uk/tools/msa/muscle/, accessed on 9 June 2018) [31]. After identifying the conserved regions, two primers were selected for PCR amplification based on those used for vipericidin sequence amplification [21] with some modifications. The forward primer (FW) was designed as 5′-AAG GGT TCT TCT GGA AGA CCT KGC TGG-3′, and the reverse primer (RV) was designed as 5′-TTA GAA GGG GAW GGW GAC CCC GAT GAC-3′. Additionally, a third primer (reverse, RV2: 5′-AAY TTC TTG AAC CGC TTG ACC C-3′) (K = G/T; W = A/T; Y = C/T) was designed based on a conserved region found in all the reported cathelicidins of South American viperids to confirm the identity of the amplified product. The properties and characteristics of the primers were evaluated using the Oligo Analyzer server (Integral DNA Technologies, IDT^®^, Coralville, IA, USA) (https://www.idtdna.com/calc/analyzer, accessed on 29 October 2018). A touchdown PCR protocol was employed, based on a previous method [21], with the following modifications: an initial denaturation step of 3 min at 95 °C, followed by ten cycles (30 s at 95 °C, 40 s at 66 °C (decreasing 1 °C per cycle), 60 s at 72 °C), and 20 cycles (30 s at 95 °C, 40 s at 56 °C, 60 s at 72 °C), concluding with a final extension at 72 °C for 15 min. As a control, specific primers for the *actin* gene, as previously reported, were used [19]. The amplified products were analyzed by electrophoresis on a 2% agarose gel stained with GelRed (Biotium, Hayward, CA, USA). The bands of interest (500–600 bp) were excised, and the DNA was purified using the QIAquick Gel Extraction Kit (QIAGEN, Hilden, Germany). The purified samples were sent for commercial sequencing at the UNAM Institute of Biotechnology (IBT) in Morelos, Mexico. The received sequences were edited and analyzed using the BioEdit program, version 7.7 [32] and subsequently subjected to a BLAST search to confirm their identity.

To facilitate cloning of the PCR product, two additional primers were designed: FW2: 5′-GAC GAG GAG GTG GAG AAG GAG AAG G-3′ and RV3: 5′-CCT TCT CCT TCT CCA CCT CCT CGT C-3′. These primers were used in conjunction with the first set of primers mentioned earlier. The PCR products were cloned using the TOPO^®^ TA Cloning^®^ Kit for Sequencing (Invitrogen^TM^, Carlsbad, CA, USA) and transformed into One Shot^®^ TOP 10 cells (Invitrogen^TM^, Carlsbad, CA, USA). Plasmid DNA was extracted using the Wizard^®^ Plus SV Mini-preps DNA Purification System (PROMEGA, Madison, WI, USA) following the manufacturer’s protocol. The plasmid DNA was sent for sequencing at the IBT of the UNAM, using the T7 primer as the forward primer and the M13 primer as the reverse primer. Two sequences were obtained, one with a length of 161 bp and the other with a length of 428 bp. These sequences were then combined and analyzed as a single sequence.

### 2.4. Bioinformatics Analysis

The sequence was examined and edited using the BioEdit software [32]. To confirm its identity, a BLAST analysis (https://blast.ncbi.nlm.nih.gov/Blast.cgi, accessed on 7 December 2018) was conducted. Open reading frames (ORFs) were predicted using the Translate Tool servers provided by ExPASy (https://web.expasy.org/translate/, accessed on 7 December 2018) and EMBOSS Transeq (https://www.ebi.ac.uk/Tools/st/emboss_transeq/, accessed on 7 December 2018) [33]. A multiple alignment of the amino acid sequences of the CRAMPs from snakes was performed using the Clustal Omega server at EMBL-EBI (https://www.ebi.ac.uk/Tools/msa/clustalo/, accessed on 7 December 2018) [34].

Protein domain prediction was conducted using the SMART tool (http://smart.embl-heidelberg.de/, accessed on 8 December 2018) [35] and the Pfam database (https://pfam.xfam.org/, accessed on 8 December 2018) [36]. An alignment of the conserved domain was performed using Clustal Omega and visualized using MView [37] (https://www.ebi.ac.uk/Tools/msa/mview/, accessed on 8 December 2018). The signal peptide was predicted using SignalP (http://www.cbs.dtu.dk/services/SignalP/, accessed on 8 December 2018). The cleavage site of the mature peptide and the region with anti-microbial activity in the sequence was determined using the PeptideCutter tool by ExPASy (https://web.expasy.org/peptide_cutter/, accessed on 8 December 2018) [38] and the Anti-microbial Sequence Scanning System (AMPA) (http://tcoffee.crg.cat/apps/ampa/do, accessed on 9 December 2018) [39,40], respectively. A 23-amino-acid fragment derived from the peptide was selected using the CAMP_R3_ (Collection of AMPs) software (http://www.camp3.bicnirrh.res.in/predict_c/, accessed on 9 December 2018) [41] to evaluate its activity, considering the results obtained from the four available algorithms. Additionally, the arrangement of amino acids in the α-helix configuration and the physicochemical characteristics of both peptides were calculated using ExPASy’s ProtParam Tool (https://web.expasy.org/protparam/, accessed on 9 December 2018) [38], INNOVAGEN’s Peptide Property Calculator (https://pepcalc.com/, accessed on 9 December 2018), and HeliQuest (http://heliquest.ipmc.cnrs.fr/cgi-bin/ComputParams.py, accessed on 9 December 2018) [42].

### 2.5. Synthetic Peptides

The synthetic peptides were commercially obtained from Peptide 2.0. They were lyophilized and had a purity of 98%. The identity of the peptides was confirmed through HPLC and mass spectrometry analysis, following the specifications provided by the company. Both peptides were synthesized as C-terminus amidated peptides. To prepare the peptides for further use, they were dissolved in sterile water to achieve a final concentration of 1 mg/mL.

### 2.6. Cytotoxicity Analysis

The assay was conducted following the MTT (3-[4,5-dimethylthiazol-2-yl]-2,5-diphenyltetrazolium bromide) reagent protocol (SIGMA, St. Louis, MO, USA). HEK293 cells were cultured in 96-well plates using Dulbecco’s Modified Eagle Medium (DMEM) (Biowest, Riverside, MO, USA) supplemented with 10% fetal bovine serum and 1% antibiotic–antimycotic (Gibco, Invitrogen, Carlsbad, CA, USA). A total of 15 × 10^3^ cells were seeded per well and incubated at 37 °C with 4.7% CO_2_. After 8 h, the medium was replaced with DMEM containing 1% fetal bovine serum, and the plate was incubated overnight.

Next, the peptides were diluted in serum-free DMEM according to the previously reported method [22] to achieve the following concentrations: 1.56, 3.125, 6.25, 12.5, 25, 50, and 100 µM. Triton 100× at 1% and cells in DMEM without treatment were used as positive and negative controls, respectively. Following a 24 h incubation period, the medium was removed, and 100 µL of MTT reagent (0.5 mg/mL) dissolved in DMEM was added to the cultures. The plate was then incubated for 4 h. Afterward, the MTT-containing medium was removed, and 100 µL of Dimethyl sulfoxide (DMSO) was added to each well. The plate was incubated at 37 °C, with stirring overnight. Absorbance was measured at a wavelength of 590 nm using a plate reader. Wells with untreated cells were used as the baseline for 100% cell viability. Each treatment was evaluated in triplicate and repeated in two independent experiments. The LD_50_ value was determined through nonlinear regression.

### 2.7. Hemolytic Activity Analysis

The hemolytic activity test was conducted following previously published protocols [43]. Male Windstar rats were used to collect blood, which was collected in a beaker containing 50 mL of Alsever’s solution [44]. The blood was then diluted and placed in tubes, followed by centrifugation at 2500 rpm at 4 °C for 4 min. The supernatant was discarded, and this washing process was repeated three times. The remaining red blood cells were resuspended in Alsever’s solution and calibrated. For 100% hemolysis, 50 µL of erythrocytes in solution was mixed with 950 µL of deionized water (resulting in an absorbance of 1.00 at a wavelength of 415 nm), while for 0% hemolysis, 50 µL of erythrocytes was mixed with 950 µL of Alsever’s solution. These values were used to create a two-point curve.

The peptides were tested at concentrations of 0.1, 1, 10, 25, and 50 µM. The samples were incubated for 30 min at 37 °C. The reaction was halted by centrifuging the tubes at 2500 rpm at 4 °C. The samples were then transferred to a 96-well microplate and read at a wavelength of 415 nm using a microplate reader.

### 2.8. Antibacterial Activity Analysis

The antibacterial activity of the peptides was determined using the microdilution method in Müeller–Hinton broth, following the guidelines outlined in the M07 and M100 manuals of the Clinical and Laboratory Standards Institute [45,46]. The peptides and antibiotics were tested at concentrations ranging from 0.125 to 256 µg/mL, with ampicillin and gentamicin serving as controls.

To prepare the inoculum, the pre-inoculums were cultured on a non-selective agar plate and incubated for 18–24 h. A colony was taken from these plates and diluted in saline solution (0.85%). The turbidity of the suspension was adjusted to 0.5 of the McFarland standard. In a 96-well microplate, 50 µL of the peptide or antibiotic solution was added to each well. Subsequently, 50 µL of the bacterial inoculum with an approximate concentration of 5 × 10^5^ CFU/mL was added to each well. Sterile broth alone and sterile water with the bacterial inoculum were used as controls. The experiment was performed in triplicate and repeated in two independent experiments.

The microplate was incubated at 37 °C for 20 h. After incubation, the absorbance of each well was measured at a wavelength of 655 nm using a microplate reader. The minimum inhibitory concentration (MIC) was determined as the lowest peptide concentration that inhibited visible bacterial growth.

## 3. Results

### 3.1. Crotalus aquilus Expressed a Cathelicidin Gene

**Figure 1:**
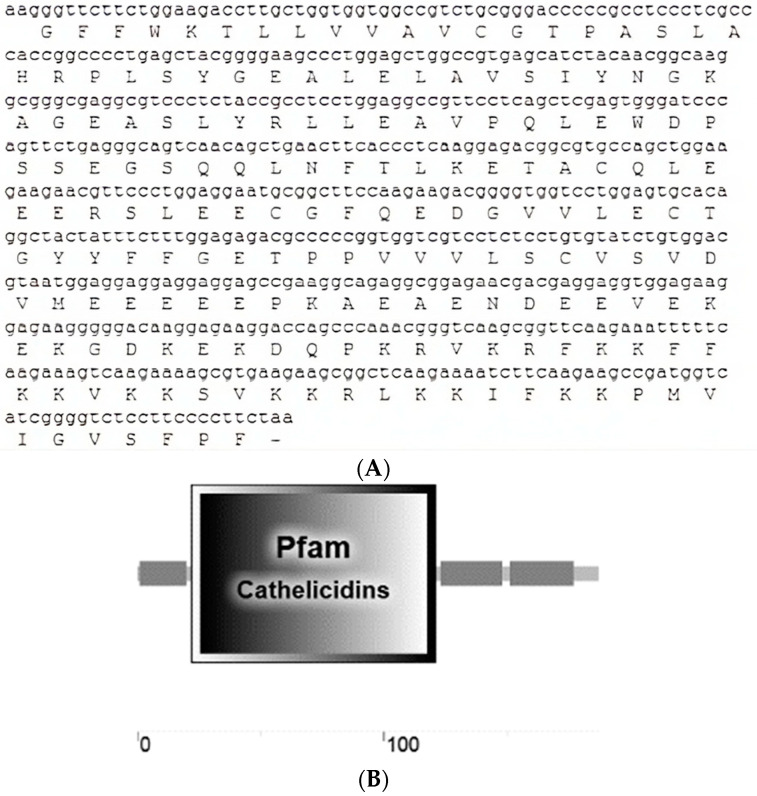
(**A**) Identification of the open reading frame in the Translate server. The nucleotide sequence is shown above the amino acid sequence, indicating the codons corresponding to each amino acid; the dash represents the stop codon. (**B**) The result of the domain prediction by the SMART server, showing the one belonging to the cathelicidin family (Pfam).

**Figure 2:**
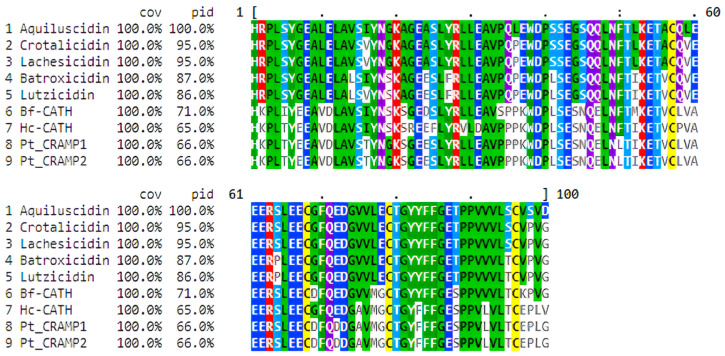
Alignment of the cathelin domain of cathelicidins identified in different snake species obtained in Clustal Omega and visualized in MView. Coverage (cov) and identity (pid) percentages are indicated in comparison to Aquiluscidin. Amino acids are shown in colors.

Sequence analysis of the cloned cDNA amplicon revealed a 566 bp sequence corresponding to a *C. aquilus* cathelicidin gene. This nucleotide sequence contained a partial ORF predicted to encode a peptide precursor consisting of 187 amino acids (Figure 1A). A 20-amino-acid-long signal peptide was confirmed at the N-terminal using both SignalP and SMART tools. Additionally, the cathelin domain was identified through Pfam and SMART tools. This domain spans from amino acids 22 to 121 of the pro-peptide (Figure 1B). When compared to cathelicidin domains from other snake species, this region exhibited a high degree of similarity ranging from 66% to 95% (Figure 2). The cathelin domain of this peptide precursor contains four highly conserved cysteines, which is a characteristic feature of cathelicidin precursors. Adjacent to the domain, a 29-amino-acid anionic region was identified (Figure 1B).

**Figure 3:**
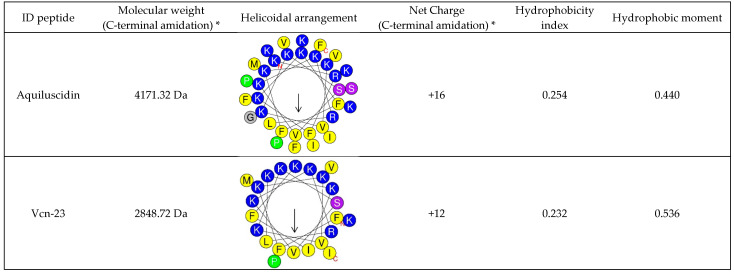
α-helix conformation predicted for *C. aquilus* cathelicidin on the HeliQuest server. Basic amino acids are shown in blue, nonpolar amino acids in yellow, and polar amino acids in purple; the arrow indicates the hydrophobic side of the helix. * Determined in Peptide Property Calculator.

The mature peptide was predicted using the AMPA and PeptideCutter tools, which identified the region responsible for the anti-microbial activity and the predicted cleavage site, respectively (Figure 1B). Both analyses confirmed the presence of valine at position 153 as the putative cleavage target for neutrophil elastase, with the potential anti-microbial zone spanning from position 153 to 183 of the full sequence. The results from both analyses were consistent and served as a reference to identify the mature peptide, which we named Aquiluscidin. Aquiluscidin consists of 34 amino acids: KRFKKFFKKVKKSVKKRLKKIFKKPMVIGVSFPF. The peptide carries a net charge of +15 due to the presence of basic residues and the absence of acidic residues. HeliQuest analysis predicts that Aquiluscidin adopts an α-helix conformation, as illustrated in Figure 3.

A shorter derivative peptide was designed and chemically synthesized as FFKKVKKSVKKRLKKIFKKPMVI-amidated. The physicochemical properties of this peptide are depicted in Figure 3. This sequence is composed of a conserved region found in both Vipericidins and Aquiluscidin, and it was selected after screening the results obtained from CAMP_R3_, which identified regions with potential anti-microbial activity. The screening process involved analyzing the predicted properties of the peptides and comparing them to those reported for AMPs with known effectiveness.

### 3.2. Cytotoxic and Hemolytic Effects

**Figure 4:**
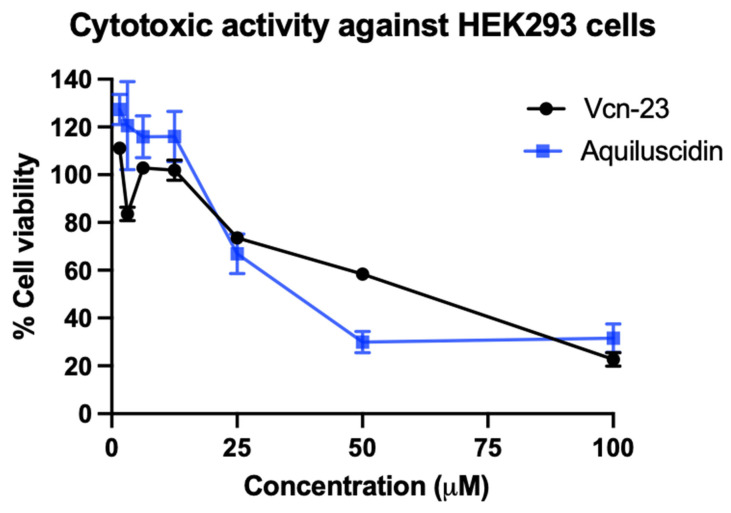
Result of the cytotoxicity assay of cathelicidin peptides in HEK293 cells, showing the different concentrations used in µM range and the percentage of viable cells corresponding to each treatment. The mean and bars corresponding to the standard error of triplicates are shown.

A cytotoxic assay was conducted using HEK293 cells and various concentrations of peptides (ranging from 1.56 to 100 µM). Aquiluscidin and Vcn-23 exhibited no toxicity at low concentrations, maintaining 100% cell viability up to 12.5 µM. However, as the peptide concentration increased, the percentage of viable cells decreased, indicating a dose-dependent cytotoxic effect (Figure 4). After treatment with 25, 50, and 100 µM with Aquiluscidin, the percentages of viable cells were 66.98%, 29.92%, and 31.51%, respectively. For Vcn-23, the values were 73.56%, 58.37%, and 22.70% at the same concentrations, The lethal dose 50 (LD_50_) for Aquiluscidin was determined to be 26.31 µM, while for Vcn-23, it was 56.27 µM.

**Figure 5:**
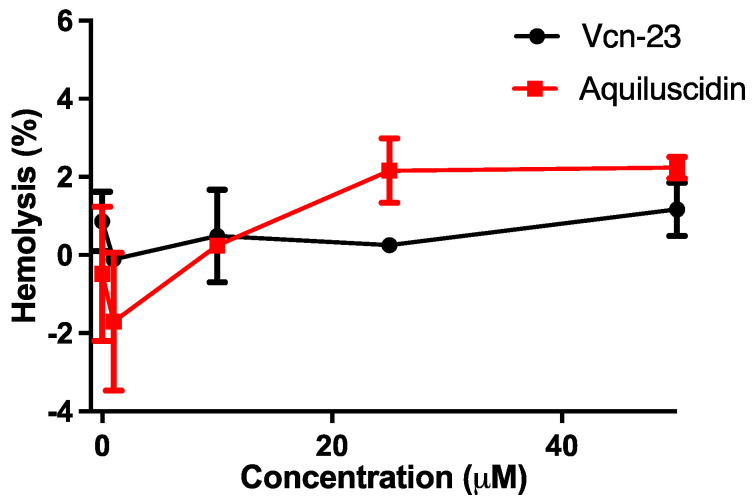
Hemolytic activity of peptides with rat erythrocytes. Different concentrations (µM) were evaluated. The black line corresponds to Vcn-23; the red line to Aquiluscidin. The mean and bars corresponding to the standard error of triplicates are shown.

In assessing the hemolytic effect, both peptides were tested at various concentrations (0.1, 1, 10, 25, and 50 µM) using rat erythrocytes diluted in Alsever’s solution. Neither Aquiluscidin nor Vcn-23 exhibited significant hemolytic activity (Figure 5). Aquiluscidin resulted in 2.16% and 2.23% hemolysis at concentrations of 25 and 50 µM, respectively. On the other hand, Vcn-23 caused 0.25% and 1.17% hemolysis at the same concentrations (Figure 5).

### 3.3. Antibacterial Activity

The anti-microbial activity of the peptides was assessed against nine different strains of bacteria belonging to six species using the broth microdilution test. The summarized results in Table 1 indicate that both peptides exhibited activity against all tested Gram-positive and Gram-negative bacteria. Aquiluscidin inhibited all strains at concentrations ranging from 2 to 8 µg/mL (Table 1). The Aquiluscidin peptide was most effective against the commercial strain of *E. coli* Top-10, with a MIC of 2 µg/mL. Additionally, it showed similar efficacy against five other strains: *E. coli* AC, two strains of *Pseudomonas aeruginosa*, *S. saprophyticus* ATCC BAA-750, and *E. casseliflavus* ATCC 700327, all with a MIC of 4 µg/mL. *S. typhymurium* ATCC 14028 and the two *S. aureus* strains displayed lower susceptibility to Aquiluscidin, requiring a MIC of 8 µg/mL. However, even at this concentration, Aquiluscidin demonstrated the ability to affect their growth (Table 1).

In contrast, the Vcn-23 peptide inhibited bacterial growth at concentrations of 2–4 µg/mL for all evaluated strains. The strains *S. saprophyticus* ATCC BAA-750, *S. typhymurium* ATCC 14028, *E. casseliflavus* ATCC 700327, and the laboratory strain of *E. coli* exhibited the lowest susceptibility, requiring only 2 µg/mL of Vcn-23 to inhibit their growth. Notably, the clinical isolates of *P. aeruginosa* and *E. coli* showed resistance to ampicillin (MIC = 256 µg/mL); however, both peptides demonstrated inhibitory activity at a concentration of 4 µg/mL (Table 1).

## 4. Discussion

When immune cells find a pathogen or a cytokine stimulus, they release different protective substances. Some of these substances act as chemical barriers to stop the infection process. AMPs are among these defense mechanisms. They are small, amphipathic, cationic molecules primarily composed of lysine and arginine and have been identified in a wide range of species [12,47].

HDPs, also referred to as AMPs, can be synthesized by epithelial cells. In organisms lacking an evolved adaptive response, their activity is crucial in inhibiting the growth of pathogens. The functional properties of AMPs are determined by their structural characteristics. In addition to their direct microbicidal action, these molecules possess diverse immune-response-modulating functions [15,48,49]. Among the animal-derived AMPs that have been studied, snake peptides exhibit a broad spectrum of activity against bacteria, fungi, and parasites, even at low micromolar concentrations [18,21,26].

Using mRNA isolated from the oral mucosa and skin of *C. aquilus*, we discovered a 566 bp transcript that encodes a peptide showing significant similarity to previously reported cathelicidin-like peptides found in snakes (approximately 95% identity with vipericidins: crotalicidin and lachesicidin) [21]. Snake cathelicidin transcripts generally range in size from 555 to 585 bp, highlighting the substantial similarity in size of this gene with those of closely related species [19,21,22,23]. The same finding was observed through ORF prediction, which revealed a putative pre-pro-peptide consisting of 187 amino acids (likely 189 residues in the complete sequence, accounting for the absence of the first two amino acids at the N-terminus). Pro-peptides of cathelicidin identified in other snake species typically consist of 184 to 194 residues [19,21,22,23].

Cathelicidin precursors possess a signal peptide at the N-terminus, which is typically composed of 20–30 amino acids. The cleavage site is found between proline/alanine and histidine residues [21,50]. In the Aquiluscidin pre-pro-peptide, a 20-amino-acid signal peptide was identified in the N-terminal region, with the putative cleavage site occurring between alanine 20 and histidine 21 residues. Cathelicidin pro-peptides contain a conserved cathelin domain [12], consisting of 101 amino acids in humans [50]. The predicted *Crotalus aquilus* cathelin domain was found between the range of amino acids 22 to 121. This region, comprising 100 amino acids, exhibits a similarity ranging from 66% to 95% with cathelicidin pro-peptides from viperids and elapid snakes. Notably, four conserved cysteines are present in this region, as described in mammals, reptiles, and cathelicidins of other species [19,21,22,23]. Previous studies have reported the presence of an anionic region in cathelicidin precursors from amphibians and reptiles, which is believed to be involved in the folding process and neutralization of the net charge [19,21,51,52]. Similarly, the Aquiluscidin pro-peptide predicted in this study also exhibits a glutamate-rich region between the cathelin domain and the cleavage site of the mature peptide, spanning from position 121 to 149.

Bioinformatics analysis predicted that the mature peptide, responsible for the anti-microbial activity, would begin after cleavage at valine 153, releasing an active 34-amino-acid molecule. Valines are known targets for elastases, the primary enzymes involved in cathelicidin processing, and this conserved site has been observed in peptides from various animal species [13,53]. Thus, we utilized the neutrophil elastase configuration to predict the cleavage region, as this approach has been previously employed to predict cleavage sites in peptides with a high degree of similarity to the one reported here [18]. Aquiluscidin carries a net charge of +15, which increases to +16 when synthesized with C-terminal amidation. This modification has been used with other peptides to protect against degradation, increase the antimicrobial activity, and help to adopt the α-helical conformation [49,54,55]; these are the reasons why we decided to synthesize the peptides with C-terminus amidated. HeliQuest prediction indicates that Aquiluscidin adopts an α-helical conformation. The peptide’s hydrophobic moment was calculated to be 0.440, and its hydrophobicity index was measured at 0.254. These characteristics influence the peptide’s efficacy and cytotoxic potential [56], and in this case, they resemble those reported in other peptides with significant anti-microbial effects [17,21]. In antibacterial assays, Aquiluscidin demonstrated high effectiveness against all tested strains at concentrations ≤1.91 µM. Similar to some cathelicidins identified in South American viperids and Asian elapids, Aquiluscidin required a higher concentration to inhibit the growth of Gram-positive bacteria compared to most Gram-negative strains, except *S. typhymurium* ATCC 14028 [19,21,22].

Due to the high cost of using chemically synthesized peptides for treatment, there has been a need to identify derivatives based on shorter molecules that can exhibit the same efficacy as native peptides [17,57]. In order to engineer a smaller sequence that is predicted to have significant anti-microbial properties, a conserved region between Aquiluscidin and two Vipericidins called Vcn-23 was selected [21]. This fragment spans from phenylalanine 6 to isoleucine 28 of the mature Aquiluscidin, and no previous evaluation of the anti-microbial potential of this sequence has been reported. Vcn-23 demonstrated inhibitory effects on bacterial growth in a concentration range of 0.70–1.40 µM, and it was particularly effective against *S. typhymurium* ATCC 14028, *E. casseliflavus* ATCC 700327, *S. saprophyticus* ATCC BAA-750, and the two *S. aureus* strains compared to Aquiluscidin. These results can be attributed to the design of Vcn-23 and the criteria used for selecting this specific region. Vcn-23 shares certain physicochemical characteristics with two elapid cathelicidins (BF-CATH and Hc-CATH) that have demonstrated significant antibacterial activity [19,22]. Additionally, like Aquiluscidin, Vcn-23 has an amidated C-terminus, which increases its cationic properties.

When evaluating the potential anti-microbial effects of small peptides, four crucial characteristics have been previously identified: the α-helix configuration, net charge, hydrophobic moment, and hydrophobicity index [56]. Based on the anti-microbial activity of other peptides [18,19,22,57], the most significant physicochemical parameters were determined as follows: a propensity to form a well-defined helical structure with distinct polar and nonpolar sides, a net charge of ≥+10, a hydrophobic moment of ≥0.420, and a hydrophobicity index of ≥0.220. Modifications in these values during the design of derived peptides often lead to a decrease in their anti-microbial effects. Vcn-23, as predicted by bioinformatics, exhibits a helical structure formed by the first 15 N-terminal amino acids, while the last 7 amino acids adopt an unstructured configuration. As shown in Figure 2, it displays a well-defined hydrophobic face and values close to the mentioned parameters in terms of the hydrophobic index (0.232), net charge (+12), and hydrophobic moment (0.536). Other snake cathelicidins with pronounced anti-microbial activity, resistance to degradation when incubated with serum, and limited cytotoxic and hemolytic capacity exhibit similar parameters [19,22,57].

An important aspect to highlight is that Vcn-23 exhibited comparable efficacy to its parent peptide (Aquiluscidin), yielding similar results at each concentration employed in the inhibition assay. Moreover, while Aquiluscidin only demonstrated significant cytotoxic potential at concentrations higher than 12.5 μM and did not exert any hemolytic activity against mammalian cells, Vcn-23 was found to be slightly less harmful after both assays. This is one of the main advantages that derivative peptides have when compared with their parental peptides [18].

The results obtained in the cytotoxicity assay are consistent with those previously published when evaluating the cytotoxic activity of different cathelicidins and their derived peptides against various cell lines. Such is the case of crotalicidin (Ctn), which exhibited strong cytotoxic activity against fibroblasts (IBR3G), with an LD_50_ of only 6.25 µM, while its derivatives Ctn [1,2,3,4,5,6,7,8,9,10,11,12,13,14] and Ctn [15,16,17,18,19,20,21,22,23,24,25,26,27,28,29,30,31,32,33,34] were practically non-toxic at the highest evaluated concentration, i.e., 100 µM [18]. The potential cytotoxicity of the same cathelicidin and its two fragments against human kidney cells (HK-2) resulted in a lower effect by Ctn [1,2,3,4,5,6,7,8,9,10,11,12,13,14], with an LD_50_ of 400 µM, while for Ctn [15,16,17,18,19,20,21,22,23,24,25,26,27,28,29,30,31,32,33,34] and Ctn, it was 50 and 3.12 µM, respectively [25]. These data demonstrate that the cytotoxic potential of each peptide varies depending on the type of cells against which they are evaluated, and it is also evident that the doses at which cytotoxic activity occurs are modified when the evaluated peptide is fragmented, due to the variation in its physicochemical characteristics that influence its biological activity [56]. Furthermore, the selectivity of peptides and the assessment of their cytotoxic effects depend on various factors, such as the quantity of cells used in in vitro assays. For instance, antimicrobial peptides typically exhibit reduced hemolytic activity due to the high concentration of erythrocytes used in the experiments. Another factor to consider is that several cell types may express certain sulfated glycosaminoglycans, which carry a negative charge that could serve as an electrostatic attractor for cationic peptides (reviewed in [58]). Conversely, in the case of red blood cells, the peptide’s hydrophobicity plays a more significant role in exerting a hemolytic effect. A higher degree of hydrophobicity is associated with increased erythrocyte lysis (reviewed in [49]). These peptides possess a moderate level of hydrophobicity, which is ideal for maintaining antimicrobial activity without being harmful to red blood cells [49,56]. This may explain why our molecules, Aquiluscidin and Vcn-23, demonstrated different performances in the cytotoxicity and hemolysis evaluation.

In conclusion, we first identified a cathelicidin-like peptide in an endemic species of the Mexican rattlesnake, *Crotalus aquilus*. The mature 34-amino-acid peptide exhibited anti-microbial activity against both Gram-positive and Gram-negative bacteria. Furthermore, we assessed the effectiveness of Vcn-23, a derivative peptide, which displayed anti-microbial activity comparable to that of complete cathelicidins. This finding is particularly significant as Vcn-23 is a smaller molecule, making it easier and more cost-effective to produce. These results indicate the considerable functionality and utility of these peptides, not only as a foundation for designing new therapeutic agents but also as direct compounds with antibacterial properties to be used in the treatment of infectious diseases, including those caused by antibiotic-resistant microorganisms. Further experiments are needed to evaluate the activity of these peptides against other types of pathogens to assess their true anti-microbial capacity.

## Figures and Tables

**Table 1:** Antibacterial activity of Aquiluscidin, Vcn-23, and antibiotics.

Anti-Microbial MIC ^+^ µg/mL (µM)				
Microorganism	Aquiluscidin	Vcn-23	Ampicillin	Gentamicin
*E. coli* TOP10	2 (0.47)	2 (0.70)	1 (2.86)	0.125 (0.26)
*S. aureus* ATCC6538	8 (1.91)	4 (1.40)	0.5 (1.43)	0.125 (0.26)
*P. aeruginosa*	4 (0.95)	4 (1.40)	1 (2.86)	0.125 (0.26)
*E. coli* CI *	4 (0.95)	4 (1.40)	256 (732.67)	0.125 (0.26)
*S. aureus* CI *	8 (1.91)	4 (1.40)	0.125 (0.35)	0.125 (0.26)
*P. aeruginosa* CI *	4 (0.95)	4 (1.40)	256 (732.67)	0.25 (0.52)
*S. saprophyticus* ATCC BAA-750	4 (0.95)	2 (0.70)	1 (2.86)	0.125 (0.26)
*S. typhymurium* ATCC 14028	8 (1.91)	2 (0.70)	0.5 (1.43)	0.25 (0.52)
*E. casseliflavus* ATCC 700327	4 (0.95)	2 (0.70)	0.125 (0.35)	1 (2.09)

^+^ MIC. Minimal inhibitory concentration. * CI. Clinically isolated strain.

## Data Availability

Data is contained within the article.
